# Establishing research priorities for malaria elimination in the context of the emergency response to artemisinin resistance framework-the Cambodian approach

**DOI:** 10.1186/s12936-016-1117-9

**Published:** 2016-02-25

**Authors:** Sara E. Canavati, Harriet L. S. Lawford, Bayo S. Fatunmbi, Dysoley Lek, Narann Top-Samphor, Rithea Leang, Arjen M. Dondorp, Rekol Huy, Walter M. Kazadi

**Affiliations:** Department of Clinical Tropical Medicine, Faculty of Tropical Medicine, Mahidol University, 420/6 Rajvithi Road, Rajthevee, Bangkok, Thailand; The National Center for Parasitology, Entomology and Malaria Control, Ministry of Health, Phnom Penh, Cambodia; Emergency Response to Artemisinin Resistance, WHO Representative Office in Cambodia, Penh Phnom Penh, Cambodia; Mahidol Oxford Tropical Research Unit, Faculty of Tropical Medicine, Mahidol University, Bangkok, Thailand

**Keywords:** Malaria elimination, Cambodia, Operational research, Artemisinin resistance

## Abstract

**Background:**

Countries of the greater Mekong subregion have made a transition from malaria control to an aim for falciparum and vivax malaria elimination. The elimination of falciparum malaria will have to be achieved against a background of increasing artemisinin and multi-drug resistance. This ambitious goal requires an operational research (OR) agenda that addresses the dynamic challenges encountered on the path to elimination, which will need to be flexible and developed in close relation with the cambodian national programme for parasitology, entomology and malaria control (CNM). In Cambodia, a number of meetings with stakeholders were convened by the CNM and emergency response to artemisinin resistance (ERAR) hub, producing an initial list of priority OR topics. The process and outcome of these meetings are described, which could serve as a template for other countries in the region.

**Methods:**

A landscaping exercise was conducted to gather all past, on-going and planned malaria focussed OR activities conducted by the cambodian research consortium in Cambodia and categorized according to research theme. The six themes included (1) malaria epidemiology, surveillance and response, (2) malaria case management, (3) malaria vector control, (4) malaria behavioural issues, (5) malaria clinical studies, and (6) other vector-borne diseases (dengue, neglected tropical diseases, soil-transmitted helminths). The different themes were discussed in small focus groups, which made an initial prioritization list which was then presented to a plenary group for further discussion. This produced a list of research questions ranked according to priority.

**Results:**

OR priorities produced by the thematic groups were discussed in the plenary meeting and given a priority score by group voting. A list of 17 OR questions were developed, finalized and listed, which included questions on surveillance, active case detection and treatment efficacy.

**Conclusion:**

This paper describes ERAR’s work on supporting Cambodia’s transition to malaria elimination by identifying national operational research priorities. ERAR has initiated and currently plays a critical role in the development of country specific research agendas for malaria elimination. The first example of this has been the described exercise in Cambodia, which could serve a template for setting OR priorities in the wider region.

## Background

In the past decade, large-scale funding and the development of improved technologies and strategies have resulted in impressive improvements in malaria control, and this in turn has led to renewed attempts at malaria elimination in a number of countries [1]. All countries in the greater Mekong sub-region (GMS) have set national malaria elimination goals. At the same time, global and regional strategic support for these efforts is growing, evidenced by the World Health Organization’s (WHO) new Global Technical Strategy for Malaria 2016–2030 [2] and a strategy for malaria elimination in the Greater Mekong sub-region 2015–2030 [3], as well as the newly defined Asia Pacific regional elimination goal of 2030 [4].

In Cambodia, there has been a marked decrease of 81 % in annual cases due to *Plasmodium falciparum* since 2009 [5]. Cambodia is now moving towards elimination, and aims to move towards pre-elimination of malaria with special efforts to (1) contain artemisinin-resistant *P. falciparum* malaria by 2015, (2) to achieve elimination of falciparum malaria (and thus malaria deaths) by 2020, and (3) to achieve a phased elimination of all forms of malaria in Cambodia by 2025 [6]. To achieve these elimination targets, Cambodia must increase and accelerate operational research (OR) activities around malaria elimination.

As countries approach elimination status, there is a real and documented risk of resurgence [7] which has been linked to the weakening of national programmes and accompanying resource constraints when malaria disappears as a public health threat [7]. Cambodia faces the additional important challenges of artemisinin resistance (ART-R) and multi-drug resistant falciparum malaria causing high treatment failure, which threatens to hamper the progress made in reducing the number of malaria cases. In addition, Cambodia has highly porous borders [8] and highly mobile populations [9], increasing the risk of imported cases from neighbouring malaria endemic countries. Documented evidence of poor anti-malarial drug quality also risks further impeding the development of resistance by increasing drug pressure on partner drugs [10, 11].

The emergency response to artemisinin resistance in the greater Mekong sub-region (ERAR) is a regional framework for action that was launched by the World Health Organization (WHO) in April 2013, with an aim to scale up malaria intervention and containment efforts in the Greater Mekong sub-region (GMS) [12]. This framework ensures both a coordinated response to ART-R in the region and continued motivation for stakeholders and national programmes to continue their efforts to achieve malaria elimination, whilst also contributing to the development of a regional OR agenda for elimination in the specific context described above.

OR can be defined as: ‘*the search for knowledge on interventions, strategies or tools that can enhance the quality, effectiveness, or coverage of programmes in which research is being done*’, hence OR should contribute to either influencing policy change or improving performance at a district, national or international scale [13]. OR activities are encouraged by donors and development partners in order to tackle obstacles relating to scaling up projects; in particular the global fund to fight aids, tuberculosis and malaria (GFATM) recommends their projects to spend 5–10 % of their budget on monitoring and evaluation, including OR [14]. Engaging stakeholders and policy and decision-makers when developing research questions can help increase acceptance, collaboration and ownership of activities by ensuring interaction between stakeholders on the research and decision-making sides, and encouraging mutual trust and learning [15–19].

The WHO has emphasized the importance of OR as more countries move towards malaria elimination, and setting research priorities can help orient research towards the specific needs [20–25]. The Global Plan for Artemisinin Resistance Containment (GPARC), published in 2010, recommends investing in ART-R related research [24] and Action 8 in the ERAR Framework for Action 2013–2015 stipulates the need to “*Fast track priority research and refine tools for containment and elimination*”. The framework highlights the importance of sharing and disseminating information as well as lessons learned with stakeholders both nationally and regionally [25].

To date, two meetings have been conducted on the prioritization of OR at a global and regional scale. At the WHO/GMP meeting in October 2013 in Geneva, Switzerland, participants prioritized 17 OR questions from an overall 45 questions [1] and at the SEARO/WPRO conference in December 2013 in Bangkok, Thailand, this priority list was reduced to six questions relevant to the GMS countries and a further five tentative OR questions for Cambodia specifically [26].

Cambodia has recognized the importance of OR in conjunction with elimination activities, and in October 2010 a regional meeting funded by USAID was organized around identifying and prioritizing key OR areas including prevention, case management, *Plasmodium vivax* and glucose-6-phosphate dehydrogenase deficiency (G6PD deficiency), vulnerable populations, Monitoring and Evaluation and surveillance, health systems and the private sector [27]. These research areas were highlighted by national programmes in the Mekong as critical to help countries move from control to elimination [27]. Following this, the Cambodian national programme for parasitology, entomology and malaria control (CNM) established the CNM Task Force for Research in January 2014 to develop and review the institutional research agenda for CNM, review and approve research proposals and studies prior to submission to funding agencies and oversee progress made on CNM’s research studies, evaluations and surveys. To accelerate research further, the time to obtain ethical approval for research, and the development of the OR agenda, CNM’s Sub technical working group ratified the establishment of the Cambodia research consortium (CRC) on 09 May 2014 [28].

The Ministry of Health of Cambodia, with technical assistance and support by WHO-ERAR, held a two-day workshop on the 3–4 June 2014 with the members of the CRC, which includes various national and international partners, stakeholders, NGOs and members of the Cambodian provincial health teams.

The objectives of the workshops were to:

1. Review the malaria OR landscape;
2. Identify operational challenges, bottlenecks and priority research questions in the transition from malaria control towards elimination; and
3. Reach agreement among the meeting participants on the next steps, roles and responsibilities in line with the CNM’s requirements.

This paper discusses the methods used by the Ministry of Health/Government of Cambodia and the ERAR framework to ensure effective and efficient use of OR to address its evolving malaria and other vector borne disease issues. Here it is described the process used to (1) prioritize research questions related to malaria elimination in the Cambodian context; and (2) address programme gaps.

## Methods

In order to prioritize OR questions we reviewed examples from other international and regional efforts [29–35]. Of particular use were the Planning Meeting for Operational Research on Malaria Elimination organized by the WHO/GMP and held in 17–18 October 2013 in Geneva, Switzerland [1], as well as the GMS regional meeting organized by SEARO and WPRO: ‘*Informal consultation on operational research to support accelerating malaria elimination in the context of artemisinin resistant falciparum malaria in the Greater Mekong Sub region’* in December 2013, Bangkok, Thailand [26].

Prior to the workshop on the 3–4 June 2014 a concept note was developed including a brief literature review, and a partner inventory form was completed by all research partners. The partner research inventory list 2014 is a comprehensive database of malaria research studies, which are recently finished, under implementation or are planned in the immediate future by partners in Cambodia. Every research study was classified according to the type of study and study theme. After the workshop, additional information was gathered via face-to-face interviews and follow-up emails with relevant stakeholders. During this process, participants from MoH (including central and provincial levels), NGOs, academics, the private sector, donors and policy makers were involved and actively participated in the workshops.

To identify priority malaria OR questions a three-step process was used, based on the aforementioned meeting in October 2013 in Geneva:

1. Participants were allocated to working groups under one of the six thematic areas (1) malaria epidemiology, surveillance and response, (2) malaria case management, (3) control of malaria vectors, (4) malaria behavioural issues, (5) malaria clinical studies and (6) other vector borne diseases [dengue, neglected tropical diseases (NTDs) and soil-transmitted helminths (STH)].
2. Participants were asked to develop a short list of prioritized questions in each theme;
3. Scoring across themes according to agreed prioritization criteria; and
4. Consensus on final priority list.

In order to understand the success and utility of the prioritization process, and allow qualitative evaluation to inform both Cambodia’s next steps and recommendations to other countries facing similar challenges regarding the management of OR, a second workshop was conducted using participatory approaches embedded in the sub-technical Working Group Meeting held on 17th September 2014, organized by CNM with support from ERAR–GMS.

The objectives of this evaluation were to:

1. Gain participants’ perspectives on the success and challenges of the prioritization process;
2. Identify lessons learnt for future use of this approach;
3. Discuss how to translate the outcome of a prioritized list of research questions into operation in-country with present and new stakeholders; and
4. Identify challenges that remain in supporting the translation of research into policy and practice and how to address these issues.

Participants were split into the following three groups to discuss in-depth one of the following topics linked to the objectives:

1. Comments on the process;
2. How to maintain momentum and follow through on priorities; and
3. What else is needed to translate research priorities into policy and practice?

Finally, a plenary was held with the group to discuss the outcomes and to get their feedback.

## Results

### List of prioritized questions in each theme

Within the six thematic groups, six to eight participants were required to list 10 specific research questions that addressed gaps in research, and from this list identify three to five questions that were most important. Each group then reported on the prioritized questions to plenary, which then worked to refine the questions. As a result, a total of 20 research areas were developed across the six themes (Table 1).

**Table 1:** Specific research areas identified, by thematic area

Thematic area	Research areas
Malaria epidemiology, surveillance and response	Mapping of mobile migrant populations (MMPs) (malaria burden in MMPs; Information on MMP status for every case in the MIS; prophylaxis for malaria control in MMP)
	Improved case surveillance (case-based surveillance with GIS (low endemic settings); detailed malaria risk maps; Real time cross-border surveillance to be used by both the countries; follow up surveillance for directly observed therapy (DOT) adherence)
Malaria case management	Asymptomatic cases and active surveillance and treatment
	Feasibility of conducting DOTs for malaria cases
	Use of microscopy as a primary method of laboratory confirmed diagnosis for malaria and establishment of a quality control program
Control of malaria vectors	Vector Mapping—Changing environment/Epidemiology/variation across the country requires research into vectors (changing biting patters, life-cycle, species, exo/endophilic, etc.)
	Insecticide resistance: research into potential pyrethroid resistance/insecticide efficacy
	Bed net research—Good national coverage, but how effective are they, are they protecting the most at risk populations (mobile migrant workers); Sleeping behaviour of the net use net preference studies (hammocks, texture, colour)
Malaria behavioural issues	How Does DOT change behaviour of the patients? Adherence of DOTs
	Huge investments in DOTs—are they worth it?
	How can we increase participatory village interventions? Active community involvement?
	How can we increase the malaria education provided to patients by health facilities/village malaria workers (VMW)?
	How best to communicate with MMPs/forest goers?
Malaria clinical studies	New ACTs drug efficacy, Artesunate + Pyronaridine
	First line treatment drug efficacy (*Pf, Pv*)
	Safety of primaquine (G6PD Screening)
Other vector borne diseases (Dengue, NTDs and STHs)	Dengue/chikungunya surveillance and estimation of disease burden—lacking in adult and private sector
	To determine a sustainable, effective and ecologically safe vector control measure for dengue control
	NTD surveillance and estimation of disease burden—lacking in private sector
	NTD surveillance and vector control

### Rank ordering

Based on the list generated in Step 1, a ranking exercise was conducted by all participants present. The facilitators used methods and tools developed by John Snow, Inc. (JSI) [36] which were modified for the malaria elimination focus (Table 2). Criteria were agreed by all participants before start of the further exercise.

**Table 2:** Scoring criteria for prioritization

SCORE	Minimum	Moderate	High
	**1**	**2**	**3**
Significance to malaria elimination	The OR question does not have any significance in terms of malaria elimination	The OR question is reasonably significant in terms of malaria elimination	The OR question is a priority in terms of malaria elimination
Feasibility of scaling up nationwide	The OR question does not have any potential to scale up nation wide	The OR question is likely to be scaled up nation wide	The OR question can be easily scaled up nation wide
Capacity	There is no capacity in place to implement the OR	There is some capacity in place to implement the OR	There is strong capacity in place to implement the OR
Potential for funding	The OR question could have some potential for funding	The OR question has some potential for funding	The OR question has potential for funding
Political will	There is no political will to address the OR question	There is some political will to address the OR question	There is high political committed to address the OR question
Urgency	There is no urgency to address the OR question in the context of malaria elimination	There is considerable urgency to address the OR question in the context of malaria elimination	There is extreme urgency to address the OR question in the context of malaria elimination

Ranking was conducted by individual participants voting and an overall score was calculated. With a few exceptions, there was a high level of consensus regarding the ranking priorities. However, in case of important differences in scoring, participants were given an opportunity for further discussion until a consensus was reached. This list of shared, agreed priorities is shown in Table 3.

**Table 3:** Operational research topics identified and scored

Thematic area	Operational research topic	Significance to malaria elimination	Feasibility of scaling up nationwide	Capacity	Potential for funding	Political will	Urgency	Total
1. Malaria epidemiology, surveillance and response	MMP surveillance	3	2	2	3	3	3	16
	Improved case surveillance	3	2	2	3	3	3	16
2. Malaria case management	Asymptomatic cases and active surveillance and treatment	3	2	2	3	2	3	15
	Feasibility of conducting DOTs for malaria cases	2	1	2	2	2	2	11
	Use of microscopy as a primary method of laboratory confirmed diagnosis for malaria and establishment of a quality control program	2	3	2	1	1	2	11
3. Control of malaria vectors	Vector Mapping—changing environment/epidemiology/variation across the country requires research into vectors (changing biting patters, life‐cycle, species, exo/endophilic, etc., insecticide resistance)	3	2	2	3	2	3	15
	Bed net research—good national coverage, but how effective are they, are they protecting the most at risk populations (mobile migrant workers); sleeping behaviour of the net use net preference studies (hammocks, texture, colour)	3	3	2	1	1	3	13
4. Malaria behavioural issues	How does DOTs change behaviour of the patients? Adherence of DOTs	2	3	3	2	3	2	15
	How can we increase participatory village interventions? Active community involvement such as active case detection?	3	3	3	3	3	3	18
	How can we increase the malaria education provided to patients by health facilities/VMWs?	3	3	3	2	3	3	7
	How best to communicate with MMPs/forest goers?	3	2	2	3	2	3	15
5. Malaria clinical studies	First line treatment drug efficacy (*Pf, Pv*)	3	3	3	2	3	2	16
	New ACTs drug efficacy, Artesunate + Pyronaridine	3	1	3	3	1	3	14
	Safety of primaquine (G6PD screening)	3	3	3	1	2	3	15
6. Other vector borne diseases (Dengue, NTDs, STHs)	Dengue/Chikungunya surveillance and estimation of disease burden—lacking in adult and private sector	2	2	2	1	2	3	12
	To determine a sustainable, effective and ecologically safe vector control measure for dengue control	3	1	1	1	1	3	10
	NTD surveillance and estimation of disease burden—lacking in private sector	N/ A	N/A	N/A	N/A	N/A	N/A	0
	NTD surveillance and vector control	N/A	N/A	N/A	N/A	N/A	N/A	0

Following the discussions, ‘insecticide resistance’ under the umbrella of ‘vector mapping’ was included as a research priority. Ranking was not conducted for ‘other vector-borne diseases’ as most participants were malaria experts, though this was still identified as a priority in Step 1.

### A list of priority questions

In the subsequent plenary meeting, the results of the ranking (ranging from high to lower priority) were reviewed to obtain consensus on a list of priority topics, which were then translated into corresponding research questions (Table 4).

**Table 4:** Operational research topics ranked by scoring criteria

No	OR Topic	Score
1	How can we increase participatory village interventions in active community involvement such as ACD and treatment?	18
2	How can we increase the quality and effectiveness of malaria education provided to patients by health facilities/VMWs, to improve awareness of, and prevention measures against malaria?	17
3	What are the most effective means of improving MMP Surveillance?	16
4	What are the most effective means of improving malaria case surveillance	16
5	Monitoring the efficacy of A + M for the treatment of *Pf* and *Pv* malaria cases	16
6	Evaluation of active surveillance and treatment of asymptomatic malaria cases	15
7	How has the epidemiology of vectors changed over the past 10 years?	15
8	How does DOTs change the behaviour of the patients and their adherence to anti-malarials?	15
9	How safe is primaquine administration in Cambodia, and what is the role of G6PD Screening?	15
10	Monitoring the efficacy of novel ACTs (Artesunate + Pyronaridine) in drug resistant regions of Cambodia	14
11	How effective are LLINs in protecting the most at risk populations (mobile migrant workers), and how does net preference and sleeping behaviour affect their use?	13
12	What is the prevalence of dengue and chikungunya in Cambodia?	12
13	How feasible is it to DOTs for malaria cases in Cambodia?	11
14	Is it feasible to use microscopy as a primary method of laboratory confirmed diagnosis for malaria and can a quality control program be established?	11
15	Is there a sustainable, effective and ecologically safe alternative to Abate for vector control measure for dengue control?	10
16	How can the capacity of the private sector be improved to incorporate NTD surveillance and the estimation of disease burden?	0
17	What surveillance and vector control methods are needed for NTDs in Cambodia?	0

Once the workshop was concluded, draft minutes of the meeting were circulated to all participants for feedback, which included corrections pertaining to the plenary discussions as well as corrections and additions to the Partner Research Inventory List 2014. Once all feedback had been received, a final version of the meeting minutes was circulated.

## Discussion

According to ERAR, country-specific ART-R containment objectives include ‘undertaking basic research and OR to ensure evidence-based strategies’. International agendas have been set [29–33, 37] and ERAR has initiated and played a critical role in the development of country specific agendas for malaria elimination OR. The first example of this has been the above described work in Cambodia, which has set research and development priorities to identify knowledge gaps and tools needed to move towards malaria elimination, whilst also complementing existing research agendas.

In order to enable the use of research findings, the research capacity in developing countries needs to be strengthened to both allow researchers to produce contextually relevant research and to ensure local ownership [28]. An OR landscaping exercise conducted by WHO found few OR projects conducted regionally were led by the national malaria programmes, who have intimate knowledge of the real situation and the bottlenecks in their own programmes [1]. Researchers from low and middle income countries are best placed to provide local and national policy makers with the evidence required to inform decision making in their own nations [28]. During this process in Cambodia, involving participants from all different sections in the workshops allowed a wealth of information and experiences to be shared and utilized in the decision-making process. This also started the process of addressing a challenge identified in the landscaping—that not all partners knew of each other’s work nor participated in discussing research priorities. It enabled fruitful discussions to take place to identify OR priorities and concerns as Cambodia transitions towards elimination.

The preparation work for the workshop was an important part of the process which included meetings with key stakeholders from CNM, WHO, NGOs and academics. Circulating the Partner Research Inventory List 2014 before the workshop enabled the creation of a transparent information-sharing platform, and ensured all participants started with the same information to inform their discussions and decisions at the workshop. It is essential that this list is kept updated with the recently developed Cambodia Elimination Action Framework for Malaria (2016–2020) as well as new and upcoming projects and that it remains available to all partners. The advantage of following the three-step method for prioritizing OR was that participants were able to discuss issues both in smaller groups relating to their thematic areas, as well as in plenary. The facilitators noted that this allowed more engagement by participants, with some participants feeling more comfortable to talk both in smaller groups and in familiar subject areas. The plenary sessions then allowed people to hear the opinions of others and, in a Delphi like process, review the priorities across all aspects of malaria programmes not just those of their particular thematic interest. This allowed the development of a joint and shared view of programme needs.

OR questions on ‘drug resistance’ were mentioned a few times in the specific research areas identified (Table 1). Most participants were familiar with the issue of drug resistance and most likely the questions posed in Table 1 were meant to be in the context of drug resistance. Nonetheless, more emphasis is warranted on research around this theme, including:

- surveillance of ART-R malaria (including genetic epidemiology) and how to report best and disseminate these findings;
- response dynamics once ART-R has arrived;
- implementation research around novel treatment strategies;
- community awareness of ART-R malaria;
- prevention of spread of ART-R malaria; and
- operational aspects of community engagement aspects of mass drug administration.

The idea of a ‘Partnership Agreement’ was discussed; the agreement will define the need for research to fit into the National Strategic Plan for Malaria Elimination [6], the process of sharing of results with the programme as a priority outcome of all research, and the importance of building national capacity as part of all partners work plans. This idea was supported by all. Lessons learnt from agreements such as those in sector wide approaches (SWAps) may be a useful start for drafting the plan [38]. The instrumentality of the Cambodian Research Consortium to facilitate prospective research including fomenting dialogue between research projects and the national ethics committee for health research (NECHR) was widely discussed and welcomed [28].

It was suggested more systematic data collection and data conservation with predefined methods for analysis should be developed. This could prove to be highly productive in providing evidence more rapidly, especially given that a close link between research and programmes can enable the faster application of new evidence. The Regional Artemisinin Initiative (RAI), funded by the GFATM, is focussing on enabling a much closer link between research and implementation, which implies much closer links of research groups (including academia) and implementers (“learning by doing”); however, could also have consequences for funding models. Learning by doing is strongly suggested in the emergency context of rapidly evolving ART-R malaria and provides results in a much shorter time frame than in the usual cycle of small-scale research projects.

Especially in the context of rapidly evolving multidrug resistance-malaria (MDR) malaria, the usual cycle of small-scale research project providing us with results several years later, will take a long time. More systematic data collection and data conservation with predefined methods for analysis could prove to be highly productive in providing evidence more rapidly. The close link between research and programme ensures faster application of new evidence [39].

Governance architecture for prioritization of research themes should structured in more detail; which includes: the roles of CNM, WHO, academia, NGOs and other stakeholders. This might address the need for flexibility, since the circumstances can change quite rapidly over time. A central repository of planned, on-going and completed projects has been completed for 2014. However it is suggested that this is updated every year.

**Fig. 1:**
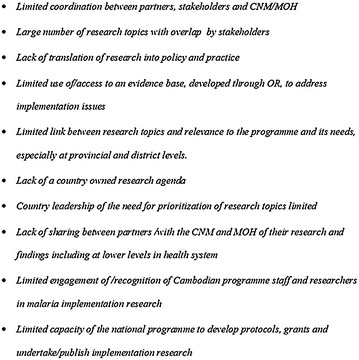
Challenges facing operational research activities in Cambodia

The process conducted in the two workshops has addressed some of the challenges identified in Fig. 1; however most participants recognized that this was not enough. A major challenge described in the landscaping that has not been fully explored to date, in the Cambodian and many similar settings, is the need for capacity development in the design, conduct, analysis and use of implementation research. Initiatives like SORT-IT [40] which have been successful in developing TB programme capacities to meet operational needs, and may be a useful approach for partners in Cambodia and the region to consider.

In addition, regional platforms like APMEN offer the opportunity for the sharing of protocols and lessons learned related to conduct operational research for malaria elimination. This sharing of real-life experience is invaluable to Cambodia when scarce scientific evidence on the topic exists to aid decision-making and can further support Cambodia to confidently develop strategies that will deliver a malaria-free Asia Pacific by 2030 [41]. Similarly, the Asia Pacific Leaders Malaria Alliance (APLMA), which is formed from the Asia Pacific Heads of Government, have agreed to the goal of an Asia Pacific free of malaria by 2030 and they have developed the Leaders’ Malaria Elimination Roadmap to establish a technically robust, strategically coherent and regionally coordinated approach to malaria elimination [42].

The malaria eradication scientific alliance (MESA) and the MESA-Track enables malaria research project to be shared worldwide [43]. Likewise the worldwide antimalarial resistance network (WWARN) is a collaborative platform generating innovative resources and reliable evidence to inform the malaria community on the factors affecting the efficacy of anti-malarial medicines [44].

As a recommended next step, the CRC should consider defining a governance architecture for the prioritization of research themes, including the roles of CNM, WHO, acadaemia, NGOs and other stakeholders. This will allow research topics to be identified and prioritized in an organized and timely process. Lastly, the CRC must be flexible; in the current environment of ART-R it is likely that the malaria epidemiology in the region will change rapidly over time.

## Conclusions

The need for high quality, useful and operationally focussed research to assist programmes in reaching global malaria targets has been clearly identified in the new strategy for malaria elimination in the Greater Mekong sub-region (2015–2030) [3], the global technical strategy for malaria (GTS) (2016–2030) [2], and the second generation Global Malaria Action Plan “Action and Investment to defeat Malaria (AIM)—for a malaria-free world” [45]. The challenge of maximizing the use of research to improve quality, coverage, effectiveness and efficiency of malaria elimination programmes is shared by many countries. The need for the populations whom all malaria partners serve to have access to an acceptable, affordable, effective malaria service must be reached, and even more urgently with the threat of artemisinin and insecticide resistance looming.

Finding a way through the complex malaria partner landscape, and ensuring country leadership and ownership and reduction of opportunity costs is a priority. The Cambodian experience described shows one country’s attempt at addressing these gaps. Helping this become a reality, and remain the norm will require honesty, integrity and goodwill of all partners and is the responsibility of all involved in research and programme management in such settings to ensure the achievement of the underlying objectives of these initiatives. The Cambodian experience can potentially become a framework that can be used in other countries aiming to transition from malaria control to elimination.
